# Self-report measurement of pain & symptoms in palliative care patients: a comparison of verbal, visual and hand scoring methods in Sub-Saharan Africa

**DOI:** 10.1186/s12955-014-0118-z

**Published:** 2014-08-02

**Authors:** David Blum, Lucy E Selman, Godfrey Agupio, Thandi Mashao, Keletso Mmoledi, Tony Moll, Natalya Dinat, Liz Gwyther, Lydia Mpanga Sebuyira, Barbara Ikin, Julia Downing, Stein Kaasa, Irene J Higginson, Richard Harding, on behalf of EURO IMPACT

**Affiliations:** European Palliative Research Center, Department of Cancer Research and Molecular Medicine NTNU, Faculty of Medicine Trondheim, Trondheim, N-7491 Norway; Department of Palliative Care, Policy, and Rehabilitation and The Cicely Saunders Institute of Palliative Care, King’s College London, London, UK; Hospice Africa Uganda, Kampala, Uganda; Palliative Medicine Unit, University of Cape Town, Cape Town, South Africa; Division of Palliative Care, Department of Internal Medicine, University of the Witwatersrand, Johannesburg, South Africa; Philanjalo Hospice, KwaZulu Natal, South Africa; Hospice Palliative Care Association of South Africa, Cape Town, South Africa; South Coast Hospice, KwaZulu Natal, South Africa; Infectious Diseases Institute, College of Health Sciences, Makerere University, Kampala, Uganda

**Keywords:** Outcomes, Africa, Assessment, Pain, Symptoms

## Abstract

**Background:**

Despite a high incidence of life-limiting disease, there is a deficit of palliative care outcome evidence in sub-Saharan Africa. Providers of end of life care call for appropriate measurement tools. The objective is to compare four approaches to self-report pain and symptom measurement among African palliative care patients completing the African Palliative Care Association African Palliative Outcome Scale (APCA African POS).

**Methods:**

Patients were recruited from five services (4 in South Africa and 1 in Uganda). Research nurses cross-sectionally administered POS pain and symptom items in local languages. Both questions were scored from 0 to 5 using 4 methods: verbal rating, demonstrating the score using the hand (H), selecting a face on a visual scale (F), and indicating a point on the Jerrycan visual scale (J). H, F and J scores were correlated with verbal scores as reference using Spearman’s rank and weighted Kappa. A Receiver Operating Characteristic (ROC) analysis was performed.

**Results:**

315 patients participated (mean age 43.5 years, 69.8% female), 71.1% were HIV positive and 35.6% had cancer, 49.2% lived in rural areas. Spearman’s rank correlations for pain scores were: H: 0.879, F: 0.823, J: 0.728 (all p < 0.001); for symptoms H: 0.876, F: 0.808, J: 0.721 (all p < 0.001). Weighted Kappa for pain was H: 0.798, F: 0.719 J: 0.548 and for symptoms: H: 0.818, F: 0.718, J: 0.571. There was lower agreement between verbal and both hand and face scoring methods in the Ugandan sample. Compared to the verbal scale the accuracy of predicting high pain/symptoms was H > F > J (0.96–0.89) in ROC analysis.

**Conclusions:**

Hands and faces scoring methods correlate highly with verbal scoring. The Jerrycan method had only moderate weighted Kappa. POS scores can be reliably measured using hand or face score.

## Introduction

In sub-Saharan Africa the incidence of life limiting diseases such as HIV and cancer presents a major clinical and public health challenge. Annually, approximately 1.2 million people die of AIDS in sub-Saharan Africa (accounting for 69% of the global HIV burden) [1] and more than 400’000 people die of cancer [2].

Palliative care for patients with progressive incurable disease and their relatives is advocated for by the World Health Organization (WHO) [3]. African providers of end of life care call for outcome measurement tools to monitor patient outcomes in clinical practice and guide clinical care [4]. In a large survey the main reason for not using outcome measures was lack of guidance and training in use of the measure and analysis of results [5]. In order to be of use, outcome measures ought to be developed and validated locally. As well as having utility in routine clinical practice, outcome measurement using validated tools is crucial to inform education, compare service models, monitor the development of palliative care, enhance its quality through clinical audit, and to ensure equity of access to good quality care [6]. Finally, they are essential in the generation of evidence, which can be considered the fifth pillar of the WHO public health palliative care strategy [7].

In order to monitor the development of palliative care, structures can be counted (e.g. numbers of hospitals) and processes quantified (e.g. referrals to a specific service); however, the most important requirement is the measurement of patient outcomes, using measures that assess if and to what extent palliative care interventions affect patients’ experience of illness or quality of life [8]. In assessing symptoms and suffering it is best to ask the patients themselves, hence patient-reported outcome measures (PROMS) are essential in palliative care [9].

The Palliative Care Outcome Scale (POS) is a patient-reported outcome measurement tool originally developed in the UK which is now used worldwide [10,11]. Based on the original POS, the African Palliative Care Association African Palliative Outcome Scale (APCA African POS) has been developed with health care providers and patients. The APCA African POS has been validated across different services in Africa [12,13], is available in local languages in east and southern Africa as well as in English, and is the most commonly used palliative care outcome tool in Africa [5].

The APCA African POS consists of 10 items; seven concern the patient and three are addressed to the family carer. The first item relates to pain in the last three days. Pain was found in the validation study to be the most prevalent symptom of patients attending palliative care services in South Africa and Uganda: 87.5% of cancer patients and 82.6% HIV patients suffered from pain [14]. Symptoms in the past three days, which are rated in the second POS item, were also highly prevalent (75.4%). In an online survey about measures in palliative care in Europe and Africa, pain was considered the most important item of the POS and symptoms the second most important [15].

In the original POS, patients report their pain or other symptoms in an ordinal scale from 0 (not at all) to 4 (overwhelming) on a paper based questionnaire. In the APCA African POS the scale was extended to 0–5, in order to use the fingers of a hand to score, a practice which was already common in clinical practice in sub-Saharan Africa. The hand scoring method allows use of the tool without paper or in situations of limited patient literacy, potential language problems or difficulty understanding the concept of a verbal rating scale. Illiteracy rates across sub-Saharan Africa range from 20% in South Africa to 60% in Uganda [16]. Illiteracy may be accompanied with innumeracy, and even in literate populations the theoretical concept of a scale cannot always be assumed to be understood. Therefore, the development of different scoring methods is an important addition for a sub-Saharan African tool, because in populations with low literacy delivering better care depends on providing communication aides [17].

To explore the utility and validity of alternative methods of scoring in this population, two further scoring methods for the APCA African POS have been developed in addition to the verbal and hand scoring methods: rating using pictures of faces, and a visual analogue rating scale using a picture of a Jerrycan. The faces scale was developed as it is widely used in paediatric medicine and may be particularly appropriate when there is difficulty comprehending the concept of a scale [18]. The Jerrycan visual analogue scale is a novel measurement scale using a picture of a familiar object commonly weighed in everyday life by many people in sub-Saharan Africa. Landmark studies in the US found that between 7 and 11% of people with acute or chronic pain were unable to complete a visual analogue scale or found it confusing [19,20], yet the utility of visual analogue scales has not been tested in an African population with serious illness.

The aim of the study is to compare these four rating methods (visual (V), hand (H), faces (F) and Jerrycan (J)) in terms of their acceptability, agreement and accuracy in predicting high scores; to determine whether these methods can be used interchangeably; and to identify which method(s) should be used in research and clinical practice in sub-Saharan African palliative care.

## Methods

This study is an analysis of cross-sectional APCA African POS data from a sample of patients with cancer or HIV at five palliative care facilities in two sub-Saharan countries (Uganda and South Africa).

### Procedure

The study was conducted in three non-profit palliative care services and one state service in South Africa, and one non-governmental hospice service in Uganda. Inclusion criteria were consecutive adult patients (at least 18 years old) with a cancer and/or HIV diagnosis under care with sufficient physical and cognitive ability to participate in interviews.

All information and consent sheets were translated from English into the local languages (Zulu, Xhosa, Luganda, Runyankole, Runyoro and Sotho). The translations were undertaken in academic departments hosting the research and topic. Assessments were conducted in local languages and digitally recorded. Research nurses entered quantitative data into purpose-designed Excel spreadsheets, subsequently imported into SPSS for analysis.

After obtaining informed consent, the research staff took demographic details (age, gender, number of dependents, and Eastern Co-operative Oncology Group (ECOG) measure of functional status [21], and in a convenience sample administered cross-sectionally the first two items from the APCA Africa POS: “Please tell me your pain during the last 3 days from 0 = no pain to 5 = worst pain” and “Have any other symptoms like sickness, coughing, diarrhoea or constipation been affecting how you feel in the last 3 days?” (0 = not at all to 5 = overwhelmingly).

**Figure 1:**
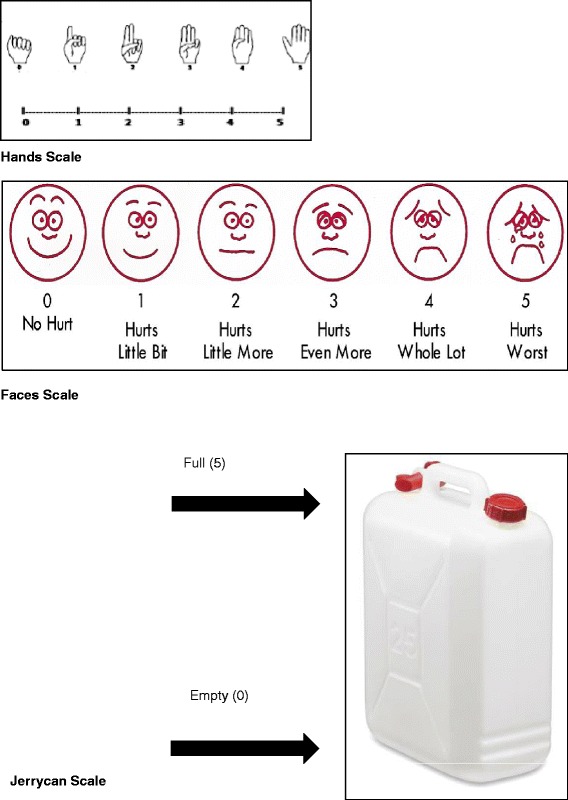
**Scales.**

Both questions were scored using the four methods for all patients (see Figure 1): 1) verbally; 2) demonstrating the score using the “hand scoring” method: closed fist = 0 (no problem) and open fingers = 5 (worst problem), with the fingers in-between representing the scores 1–4, 3) the faces visual scale (i.e. sad face for worst pain to happy face for no pain with 6 faces in total); and 4) the Jerrycan visual scale, which was constructed for the purpose of this study. The scale shows a picture of an empty Jerrycan with a verbal description of empty = no pain to full = overwhelming pain, with the respondent asked to indicate their pain level on the picture (Figure 1). A Jerrycan is a container for fuel or water. Many people in developing countries use Jerrycans to haul and store their drinking water, and it is therefore a part of everyday life for many people in Sub-Saharan Africa.

### Ethics

Ethical approval for the ENCOMPASS study was granted by the Universities of Cape Town (128/2006), KwaZulu Natal (E025/06) and Witwatersrand (M060366); the Ugandan National Council for Science and Technology (HS143), Hospice Africa Uganda; and the Hospice Palliative Care Association of South Africa (001/06).

### Analysis

The data were imported into PASW® Statistics 18 (SPSS Inc., Chicago, IL). Descriptive statistics were used to describe participants’ age, gender, primary diagnosis, functional status, place of care, and time under hospice care. To examine the distribution of the scores for the four scales, the mean and standard deviations of pain and symptom scores were calculated and graphically compared. The acceptability of the four scales was assessed by determining the rate of completion. Completion rates of the different scales were compared.

To analyse agreement between the scales, the distribution of pain and symptom scores over the different scales was measured, and the correlations between the different scales was calculated. Due to the non-parametric distributions, Spearman rank correlations were performed in order to ascertain relations between the different scales. Cohen’s weighted Kappa for agreement was calculated for all scales using the verbal scale as reference. Weighted Kappa was applied to account for the ordinal nature of the scales.

To examine differences between groups, the population was dichotomised by primary diagnosis (AIDS and cancer) and place of care (homecare vs. other). Differences in agreement between these subgroups in weighted Kappa were compared. In a second step the population was dichotomised by country: South Africa (4 services) and Uganda (1 service). Differences in agreement in weighted Kappa for the four scales were analysed. To further investigate differences in scoring methods, the population was grouped by language in which the tool was completed. The groups used were: English vs. Zulu vs. Xhosa vs. Ugandan languages (Luganda, Runyankole, and Runyoro) vs. Sotho. Agreement in weighted Kappa for the four scales was compared across the five language groups.

To determine the accuracy, sensitivity and specificity of different scales in predicting high scores, pain and symptom scores were dichotomised according to severity. High pain or high symptoms were set as cases, defined as a score of 3 or more out of 5. This was considered the clinically meaningful cut-off for high pain or high symptoms by our research team.

Scores of 0–2 on the scale were grouped as non-cases. Binary logistic regression was performed and Receiver Operating Characteristic (ROC) curves were used to investigate the sensitivity, specifity and accuracy of the new scoring systems (hand, faces, Jerrycan) to detect cases (high pain or high symptoms) compared to the verbal scoring system, which acted as reference scale in this analysis. ROC curves were graphically displayed.

## Results

### Participant characteristics

A total of 315 participants (mean age 43.5 years, 69.8% female) were included, 71.1% had HIV, 35.6% cancer and 7% had both HIV and cancer. 49.2% resided in a rural area and the majority (71%) was cared for at home. Detailed demographics are displayed in Table 1.

**Table 1:** **Sample characteristics (N = 315)**

**Age (years)**	**Mean 43.47, SD 15.459, Range 20–88**	
**Gender**	Male 30.2% (n = 95)	Female 69.8% (n = 220)
**Cancer**	Yes 35.6% (n = 112)	No 64.4% (n = 203)
**HIV**	Yes 71.1% (n = 224)	No 28.9% (n = 91)
**CA & HIV**	Yes 7.0% (n = 22)	No 93.0% (n = 293)
**Functional status (Zubrod)**	0 Fully active 21% (n = 66)	
	1 Restricted 27% (n = 85)	
	2 Ambulatory 20% (n = 63)	
	3 Limited self-care 25.7% (n = 81)	
	4 Completely disabled 6.3% (n = 20)	
**Children**	Yes 74.9% (n = 236)	No 25.1% (n = 79)
**No. of children**	Mean 2.99, SD 2.263, Range 1–11,	
**Location of home**	Urban 30.5% (n = 96)	
	Peril-urban 20.3% (n = 64)	
	Rural 49.2% (n = 155)	
**Place of care**	Homecare 71.1% (n = 225)	
	Other 28.9% (n = 90)	
	Inpatient 16.8% (n = 53),	
	Outpatient 7.3% (n = 23)	
	Day care 4.4% (n = 14)	
**Weeks under hospice (days)**	Mean 51.16, Median 19.00 SD 76.11. Range 0–488	

Each of the 5 sites recruited between 60 and 70 patients. The language the assessments were conducted in were Zulu (n = 144, 48.9%), English (n = 86, 27.3%), Xhosa (n = 27, 8.6%), Runyoro (n = 21, 6.7%), Sesotho (n = 11, 3.5%), Luganda, (n = 8, 2.5%) and Runyankole (n = 8, 2.5%). Patient and researcher fluency was 100% in the language in which the interview was conducted.

Comparison of POS scoring systems: The mean pain score was 3.07 (SD 1.54) for the verbal scale, 3.03 (SD 1.55) for the hand scale, 2.86 (SD 1.56) for the faces scale and 2.58 (SD 1.69) for the Jerrycan scale.

The mean symptom score was 3.05 (SD 1.51) for the verbal scale, 2.97 (SD 1.50) for the hand scale, 2.90 (SD 1.50) for the faces scale and 2.66 (SD 1.67) for the Jerrycan scale.

**Figure 2:**
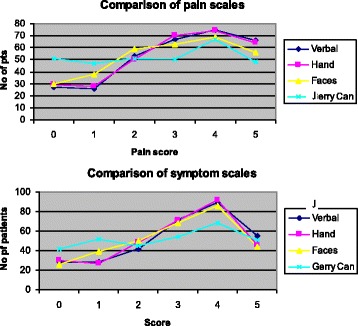
**Distributions of pain and symptom scores.**

The distribution of scores over the different scales is displayed in Figure 2.

The graph plots demonstrate that the hand, faces and Jerrycan scores diverge at the extremes of the scales and fit more closely through mid-intervals and that the verbal and hand scale scores were most closely related.

In terms of acceptability, one patient found the verbal scale difficult to use to rate pain, and one could not use the Jerrycan scale to assess pain. In assessing symptoms, one patient found both the verbal and hand scales difficult to use, two patients found the faces scale hard to use and three patients found the Jerrycan scale hard to use.

With respect to agreement, pain scale scores correlated significantly, with a correlation coefficient between 0.88 and 0.73 for non-parametric distribution (p < 0.001). Symptom scale scores also correlated significantly, with correlation coefficients between 0.88 and 0.72 (p < 0.001). Correlations are displayed in Table 2.

**Table 2:** **Spearman rank correlations between different scales**

		**Pain verbal scale**	**Pain hand scale**	**Pain faces scale**	**Pain Jerrycan scale**
**Pain verbal scale**	**r**	1	0.88	0.82	0.73
	**p**		0.001*	0.001*	0.001*
	**N**	314	314	314	313
**Pain hand scale**	**r**	0.88	1	0.85	0.76
	**p**	0.001*		0.001*	0.001*
	**N**	314	315	315	314
**Pain faces scale**	**r**	0.82	0.85	1	0.79
	**p**	0.001*	0.001*		0.001*
	**N**	314	315	315	314
**Pain Jerrycan scale**	**r**	0.73	0.76	0.79	1
	**p**	0.001*	0.001*	0.001*	
	**N**	313	314	314	314
		**Symptom verbal scale**	**Symptom hand scale**	**Symptom faces scale**	**Symptom Jerrycan scale**
**Symptom verbal scale**	**r**	1	0.88	0.81	0.72
	**p**		0.001*	0.001*	0.001*
	**N**	314	314	313	312
**Symptom hand scale**	**r**	0.88	1	0.85	0.77
	**p**	0.001*		0.001*	0.001*
	**N**	314	314	313	312
**Symptom faces scale**	**r**	0.81	0.85	1	0.82
	**p**	0.001*	0.001*	0	0.001*
	**N**	313	313	313	311
**Symptom Jerrycan scale**	**r**	0.72	0.77	0.86	1
	**p**	0.001*	0.001*	0.001*	
	**N**	312	312	311	312

*Correlation is significant at p < 0.001.

Weighted Kappa for pain score agreement with the verbal scale was as follows: 0.80 for the hand scale, 0.72 for the faces scale and 0.55 for the Jerrycan scale. For symptom score, agreement with the verbal scale was 0.82 for the hand scale, 0.71 for the faces scale and 0.57 for the Jerrycan scale.

The order of agreement (higher agreement for hand scale than faces scale, faces greater than Jerrycan scale) was consistent in the subgroups HIV and cancer. When comparing home care patients vs. other, the same results were found (hand scale > faces scale > Jerrycan scale) (Data not shown). There was less agreement of the hand scale with the verbal scale in Uganda than in South Africa (Table 3).

**Table 3:** **Subgroups: Countries Scales Agreement weighted Kappa**

**Pain**			
**KAPPA**	**Hand scale (n=)**	**Face scale (n=)**	**Jerrycan scale (n=)**
**Uganda**	0.62 (69)	0.54 (69)	0.44 (69)
**South Africa**	0.83 (245)	0.75 (245)	0.57 (244)
**Missing**	(1)	(1)	(2)
**Symptoms**			
**UGANDA**	0.71 (69)	0.56 (69)	0.56 (69)
**South Africa**	0.84 (245)	0.74 (244)	0.57 (243)
**Missing**	(1)	(2)	(3)

When analysed by language, the order of agreement (i.e. greatest for hand, hand greater than faces, faces great than Jerrycan) was congruent for all languages except for the Ugandan languages, in which there was less agreement for the faces scale than for the Jerrycan scale for both pain and symptoms. Overall agreement between the scales was lowest in the Ugandan language group when compared with the other language groups (Table 4).

**Table 4:** **Subgroups: Language Scales Agreement weighted Kappa**

**Pain**			
**KAPPA**	**Hand scale (n=)**	**Face scale (n=)**	**Jerrycan scale (n=)**
**English**	0.76 (86)	0.66 (86)	0.40 (86)
**isiZulu**	0.84 (153)	0.78 (153)	0.60 (153)
**isiXhosa**	0.77 (27)	0.65 (27)	0.44 (26)
**Ugandan**	0.57 (37)	0.44 (37)	0.50 (37)
**SeSotho**	1 (11)	0.73 (11)	0.83 (11)
**Missing**	(1)	(1)	(2)
**Symptoms**			
**KAPPA**	**Hand scale (n=)**	**Face scale (n=)**	**Jerrycan scale (n=)**
**English**	0.82 (86)	0.64 (86)	0.43 (85)
**isiZulu**	0.80 (153)	0.73 (152)	0.59 (153)
**isiXhosa**	0.85 (27)	0.77 (27)	0.61 (26)
**Uganda**	0.754 (37)	0.55 (37)	0.65 (37)
**SeSotho**	1 (11)	0.82 (11)	0.68 (11)
**Missing**	(1)	(2)	(3)

**Figure 3:**
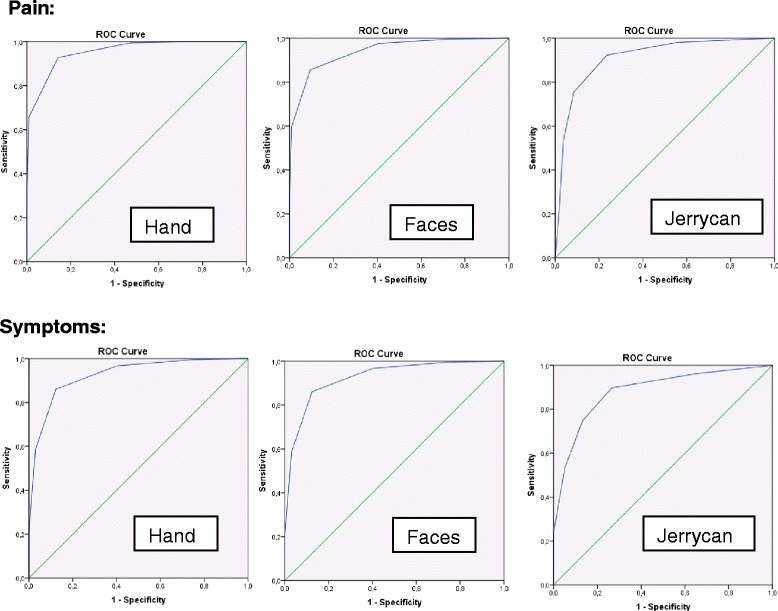
ROC Curves for pain and symptoms. **ROC Curves for pain and symptoms.** Specific coordinates: (Sensitivity/1-Specifity). Pain: Hand: (0,93/0,14) Face: (0,98/0,41); (0,86/0,09) Jerrycan: (0,92/0,24); (0,75/0,09). Symptoms: Hand: (0,97/0,45) Faces: (0,97/0,40); (0,86/0,12) Jerrycan: (0,90/0,27); (0,79/0,1).

Compared to the verbal scale, the accuracy of the other three scales of predicting high pain cases was as follows: 0.96 for the hand scale, 0.94 for the faces scale and 0.91 for the Jerrycan scale in Receiver Operating Characteristics (ROC). The accuracy of predicting high symptoms was 0.96 for the hand scale, 0,93 for the faces scale and 0.89 for the Jerrycan scale. The specific ROC Curves are displayed in Figure 3.

## Discussion

In this study, the first to compare scoring methods among sub-Saharan African palliative care patients, the hand and faces scale scores were highly correlated with verbal scores when measuring pain and symptoms. The Jerrycan scale had a high correlation but only moderate weighted Kappa when compared to the verbal scale.

Overall it is encouraging that the verbal, hand and faces scale were found to be well correlated and with high weighted Kappa values, as these are the methods for pain and symptom measurement that are currently most commonly used in African clinical and research practice. Patients completed almost all measurements; the hardest to complete was the Jerrycan scale, which 3 out of 315 patients (1%) found difficult to use to rate pain or symptoms. The results show that the hand scoring method is an acceptable and accurate scoring method compared to the verbal scoring system when administering the APCA African POS. The Jerrycan scale, which was designed for this study, was found to be the least well correlated with the verbal scale. This may be because the Jerrycan is not a calibrated measure and is different from the other scoring systems in this respect. The Jerrycan scale also does not seem to be as sensitive to detect pain as the other scales. An improvement to the Jerrycan scale as used here may be to have calibrations on the diagram or to use alternative pictures of Jerrycans of different fullness. An advantage of the hand scale could be the fact that numbers are universal in languages. Patients could be taught the assessment in their native language at first assessment, and assessed by the hand method afterwards by non-native speakers.

In the subgroup analyses the order of agreement was the same as in the whole population, except for the relatively poor agreement of the hand and faces scale with the verbal scale in Uganda. This could possibly be due to the higher illiteracy rate in Uganda (>30%) compared to South Africa (<10%), which might be accompanied with more difficulties in understanding the theoretical concept of a numerical scale.

Overall, the scales were accurate in predicting high pain compared to the verbal scale, with the hand scale (96%) more accurate than the faces scale (94%), which was more accurate than the Jerrycan (91%). This was similar for symptoms (96% vs. 93% vs. 89% respectively).

A recent systematic review examining the rating of pain intensity in adults found that numerical rating scale had better compliance in 15 out of 19 studies. Numerical rating scales were recommended in 11 of the 19 studies due to higher compliance rates, better responsiveness and ease of use, and good applicability [22]. However, a recent study in a rural Indian population, which specifically compared post-operative pain numerical rating scales and visual analogue scales in literate and illiterate populations, found no significant differences. The authors conclude that the scales can be applied interchangeably irrespective of literacy status [23]. The faces pain scale was the preferred scale in older minority adults in America, but showed lower correlations to other scales such as verbal descriptor scales, numeric rating scale or a pain thermometer, which suggests faces scales might measure a broader construct incorporating pain [24]. A study in Kenya including 15 Swahili speaking patients applying cognitive interviews showed good comprehension of numerical rating scale and faces pain scale, but a likewise clear 14:1 preference for the faces scale [25]. In this context of findings from the current study, this implies that numerical or verbal scales are most appropriate but alternative scales may be used as communication aid.

There are several limitations to our study. First, the recruitment of a convenience sample might have resulted in a bias towards higher completion rate and agreement. Second, due to procedural reasons the scales were always completed in the same order, namely number scale, hand scale, face scale and finally Jerrycan scale. This may have affected the answers given by patients; for example, patients may have calibrated their answers to the previous answer given, resulting in artificially high correlation levels, or may have lost concentration when asked the same question repeatedly, which could explain the decrease in agreement along the order of the scales. Furthermore, there was no direct measurement of literacy/illiteracy. The deduction from geographical location of the services or the language of administration is only a proxy; direct assessment of literacy is important in future research.

## Conclusion

APCA African POS scores for pain and symptoms can be reliably measured by hand scores for patients in sub-Saharan Africa compared to standard verbal measurement. The faces scale is an additional alternative with high validity. Further research is required to understand how literacy levels and linguistic and cultural differences may affect rating methods in this population.
